# Annotations

**Published:** 1906-11-03

**Authors:** 


### ANNOTATIONS.

### The Humanities and Medicine.

The relation of the classical studies to medicine must ever be in dispute. Those who look upon medicine as a trade, a mere method of making money, are apt to decry any knowledge which does not immediately and directly bear upon the business in hand. On the other hand, those who hold that medicine is a splendid profession with a great past and a more magnificent future, recognise that all studies leading to the widest education of the brain are not only permissible but necessary. But it is only the highest type of intellect which is capable of being educated on the widest lines. Now no one will seriously maintain that the higher faculties are stimulated by the half-penny newspaper, or cheap magazine in the same way as they are by reading a play of Shakespeare, a novel of Thackeray, or the lectures of Watson, Trousseau, Paget, or Pasteur. So with Latin and Greek, a boy who learns but a smattering at school may, if his brain is worth cultivating, afterwards improve his knowledge until he understands the delicacy of Horace, the ruggedness of Æschylus, and the sternness of Sophocles. Such a self-education will not only give him pleasure and lay up for him a store of interest for the leisure of his old age "in the days when the keepers of the house shall tremble, and the strong men shall bow themselves and the grinders shall cease because they are few: and those that look out of the windows be darkened"; but his breadth of outlook will thereby be increased. He will be able to take a catholic survey of disease, and as new factors present themselves to his notice he will be capable of weaving them into an hypothesis or theory which may advance materially the progress of his art. A similar amount of energy put into mathematics, or physics, or chemistry, will not produce equally good results. Literature from its dawn has dealt with man and his problems; medicine in like manner deals with mankind. Literature, therefore, may be made subservient to medicine by the best intellects, whilst science remains an ally to be cultivated assiduously but cautiously, for science has its limitations and pitfalls.

### The Moxon Medal.

The award to Mr. Jonathan Hutchinson of the Moxon Medal by the Royal College of Physicians, for "observation and research in clinical medicine," is an event of more than personal and passing interest. While offering our cordial congratulations to the recipient, it is due also to the patrons to acknowledge the informed and liberal spirit which has guided their decision. The previous Moxon medallists are Sir Alfred Garrod, Sir William Jenner, Sir Samuel Wilks, Sir Wm. Gairdner, and Dr. Hughlings Jackson — names which claim universal respect, and at the same time indicate the association of the medal with the work of the scientific physician. In a narrow technical sense Mr. Hutchinson will hardly claim this designation. He has been, as is well known, President of the Royal College of Surgeons, and for a term of 20 years or so was surgeon to the London Hospital. But his work has never been confined by those narrow and artificial lines upon which some of our contemporaries seem at the present day so anxious to insist. On the contrary, he has applied his considerable and unwearied intellectual energies to the problems of medicine in the widest sense of that term, and his achievements well entitle him to the corporate, official, and appreciative notice of the College of Physicians. But few can hope to rival either his achievement or his energy. Yet the principle of his methods may be widely commended as an example to the entire medical profession, and especially to those who are still on the threshold of their full activities. Temptations to a narrow specialism seem every day to become increasingly attractive. It seems to us, in these circumstances, all to the good to have exhibited to professional ambitions the example of a great master in whose career are portrayed not only the industry, but also the wide outlook and catholic sympathies necessary to appreciate the true meaning and inter-relationships of the problems of disease.

### The Quarrels of the Opticians.

When two separate organisations, each claiming to be the genuine Simon Pure, as the representative of one and the same constituency, awake to activity there is like to be sport for the onlooker. And even if calm existed in ordinary circumstances, it is morally sure to disappear when each organisation in its official capacity proceeds to promote a Bill in Parliament. This is exactly what has happened in connection with those two imposing bodies the Worshipful Company of Spectacle Makers and the British Optical Association. Each wishes to be recognised as a State authority for granting licences or diplomas to opticians as qualified to estimate and correct errors of refraction, and each in this capacity aspires to play the part of Codlin to the other's Short. Through the medium of rival journals the opponents have been able to announce their respective merits, and the quarrel, to quote a not unfriendly critic, has given rise to an "endless supply of controversial argument and scurrilous invective." Recently there has been some attempt to compose the issue between the disputants on the basis of a common representation on a Central Registration Board, but it is even yet not quite certain that peace will prevail. Further, the arrangement just described has called still other Richmonds into the field. The terms of the treaty appear to have excited the envy and ambitions of two organisations known respectively as the Society of Chemist-Opticians and the National Association of Goldsmiths. These, in their turn, demand the privilege of contributing representatives to that Central Board under the combined wisdom and authority of which the "prescribing optologist" hopes shortly to make his bow to the public and to offer credentials in the shape of a diploma attractively arranged in the shop window. If the quarrel now in progress succeeds in delaying this interesting appearance, our own view would be that the occasion is not one for tears.

